# Recurrent Malaria with *Plasmodium vivax*: A Case Report and Brief Review of the Literature

**DOI:** 10.3390/tropicalmed10090261

**Published:** 2025-09-12

**Authors:** Ákos Vince Andrejkovits, Adrian Vlad Pop, Magdolna Fejér, Elena Cristina Gîrbovan, Răzvan Lucian Coșeriu, Camelia Vintilă, Anca Meda Văsieșiu

**Affiliations:** 1 Department of Infectious Diseases, George Emil Palade University of Medicine, Pharmacy, Science and Technology of Târgu Mureș, 540142 Târgu Mureș, Romania; akos.andrejkovits@umfst.ro (Á.V.A.);; 2 1st Infectious Disease Clinic, Mureș County Clinical Hospital, 540233 Târgu Mureș, Romania; 3 Department of Microbiology, George Emil Palade University of Medicine, Pharmacy, Science and Technology of Târgu Mureș, 540142 Târgu Mureș, Romania; lucian-razvan.coseriu@umfst.ro (R.L.C.);

**Keywords:** *Plasmodium vivax*, recurrent malaria, case report

## Abstract

**Background:** Recurrent malaria refers to repeated episodes of the disease in the same individual. *Plasmodium vivax* is known for its ability to relapse due to dormant liver-stage hypnozoites and poses a particular risk to travelers returning from endemic areas. Prompt diagnosis and treatment are crucial to prevent recurrences. **Case Presentation:** We present the case of a 41-year-old man from Romania who developed *Plasmodium vivax* malaria after traveling through Southeast Asia without chemoprophylaxis. He presented with fever, chills, myalgia, headache, vomiting, and abdominal pain. Clinical findings included mild jaundice and slight neurological signs. Laboratory tests showed severe thrombocytopenia, elevated bilirubin, inflammatory markers, and borderline creatinine levels. Malaria was confirmed by a rapid diagnostic test and blood smear microscopy. The patient was treated with doxycycline and atovaquone–proguanil. He improved and was discharged, but experienced two relapses, both confirmed as *Plasmodium vivax* by RT-PCR. Despite receiving primaquine as radical cure after the first *Plasmodium vivax* malaria relapse, a second relapse occurred. Each episode was managed with blood-stage antimalarial therapy, leading to full clinical and biological recovery. **Conclusions:** Malaria rarely occurs in non-endemic areas; it should be considered in patients with compatible travel history and symptoms. Given the high relapse potential of *Plasmodium vivax*, accurate species identification is critical to guide appropriate long-term management.

## 1. Introduction

Malaria remains one of the most important infectious diseases globally, causing significant morbidity and mortality, particularly in endemic countries [1,2,3]. Its distribution is uneven worldwide, with the highest burden concentrated in specific regions. Sub-Saharan Africa accounts for approximately 88% of all cases, while Southeast Asia, together with Central and South America, contributes around 10% [4,5].

The disease is caused by protozoan parasites of the *Plasmodium* genus, transmitted to humans through the bites of infected *Anopheles mosquitos* [4,5]. Among the five known *Plasmodium* species that infect humans, *Plasmodium falciparum* is the most virulent and prevalent, being responsible for the majority of cases in Africa and tropical regions [6,7]. Outside Africa, *Plasmodium vivax* is the most widespread species, accounting for 80–90% of malaria cases and representing the primary cause of febrile illness in Southeast Asia, India, and the Americas [1,6,8]. Other species, such as *Plasmodium ovale* and *Plasmodium malariae*, contribute to a much smaller proportion of global cases, while *Plasmodium knowlesi* is an emerging species in Southeast Asia [3,4,6]. The geographic distribution of these species influences the clinical presentation and epidemiology of the disease, as well as the control and elimination strategies in endemic regions [7,9].

In non-endemic regions such as Europe and North America, malaria is rarely transmitted locally. Instead, it is typically an imported disease, diagnosed in international travelers and returning migrants from endemic areas [3,7]. In Europe, an estimated 7000–8000 cases are reported annually, with approximately 98–99% linked to travels to Asia and Africa [3,7,10]. *Plasmodium falciparum* is the most commonly identified species, accounting for 60–80% of cases and reflecting both travel patterns and the high transmission rates in affected regions [3,11]. The second most common species in Europe is *Plasmodium vivax* [8,10,11].

*Plasmodium vivax* is characterized by its ability to enter a dormant stage in the liver, forming hypnozoites that can reactivate weeks or even months after the initial infection. These relapses can occur repeatedly, sustaining transmission and complicating control efforts [1,12,13]. Understanding the frequency, geographic distribution, and key risk factors for relapse plays a critical role in developing effective treatment and elimination strategies.

Studies show that the frequency of relapses varies depending on the setting and population. In endemic regions such as India, Southeast Asia, and Central America, relapse rates can reach 60–80% or even higher in areas with fast-relapsing strains and limited access to radical cure—a treatment that eradicates both the circulating parasites and the dormant liver-stage hypnozoites, forms that can reactivate and later produce relapses [1,4,14]. Pooled analyses and systematic reviews have reported a wide range of relapse rates among non-immune populations, including military personnel and international travelers [13]. Detailed cohort studies indicate recurrence rates of around 9–10%, even in regions where appropriate treatment protocols are followed [2]. However, a controlled study conducted in Cambodia, in which patients were relocated to a non-endemic area, found a 60% relapse rate within 60 days among those treated with chloroquine. Once reinfection was ruled out, the findings strongly supported that, in the absence of hypnozoitocidal therapy, most recurrent *Plasmodium vivax* episodes are due to relapse [15].

Relapse patterns show a clear geographical distribution, shaped by *Plasmodium vivax* strains adapting to different climatic and transmission conditions. Studies describe three main categories: In “tropical regions”, relapses occur faster and more frequently, typically within 3–6 weeks after the initial infection [1,6,11,14]. This pattern is common in Southeast Asia, South America, and most of India [11]. In contrast, “temperate regions” are dominated by long-latency strains, where relapses can occur after 6–12 months after the primary infection. This pattern is seen in Europe, Central Asia, and North Africa [1,6,11,14]. “Intermediate regions”, such as parts of India, show both fast and long-latency relapse phenotypes coexisting, emphasizing the overlapping patterns and local variation, driven mostly by seasonality, parasite strain mix, and exposure heterogeneity [1,11,14]. In treated cohorts, the median time to relapse is around 80–90 days. Regions such as Southeast Asia and Melanesia are predicted to have the highest incidence of relapse, while Northern Asia and Europe report much lower rates [1,10,11,14].

The most important risk factors for *Plasmodium vivax* relapses include the following:

1. Parasite factors: Strain variability in relapse frequency and timing plays a major role. The size of the hypnozoite reservoir, which is closely linked to the sporozoite inoculum, is a key determinant of this risk. This inoculum size varies depending on the number and intensity of infectious mosquito bites. A large reservoir increases the likelihood of relapse [11,12,13,14].
2. Host factors: Age, acquired immunity, and treatment adherence are central to relapse risk. In addition, genetic polymorphisms can influence treatment outcomes. For example, CYP2D6 (cytochrome P450 2D6) variants may impair drug metabolism, and G6PD (glucose-6-phosphate dehydrogenase) deficiency can limit the use of hypnozoitocidal drugs due to the risk of hemolysis, leading to treatment failure. Coinfections and febrile illnesses may also act as external triggers for hypnozoite reactivation [1,4,12,14].
3. Environmental and epidemiological factors: Relapse patterns are influenced by local mosquito species, urbanization, climate, and seasonal variations [13,14].

The only effective means of preventing *Plasmodium vivax* relapses is radical cure, which targets the dormant hypnozoites in the liver. The most effective drugs currently available are primaquine and tafenoquine, both part of the 8-aminoquinoline class of antimalarials [4,14]. Primaquine remains the gold standard, while tafenoquine has shown non-inferior efficacy, with 67–74% of patients remaining relapse-free 6 months after treatment, compared to 73–76% with primaquine [4,16,17]. A meta-analysis showed that both therapies outperform placebo and standard blood-stage treatment alone—an antimalarial therapy that eliminates the circulating parasites inside the red blood cells, usually resolving the acute febrile illness [4,16]. The key differences between the two drugs lie in their administration. Primaquine requires a 14-day course with daily dosing, and medical supervision is recommended, as incomplete adherence significantly reduces effectiveness [10,12,14]. In contrast, tafenoquine is administered as a single oral dose, making it easier to use and not requiring supervision. However, it is currently approved only for adults (aged 16 and older) and is not widely available [4,16,17]. Even though tafenoquine has a more facile administration, G6PD testing is mandatory before prescription, due to the high risk of acute hemolysis in G6PD-deficient patients. Notably, due to G6PD activity being X-linked, different tests should be performed in males and females. In males, a qualitative test is sufficient to identify the deficiency. In females, due to X-chromosome inactivation, the enzyme has variable activity and a quantitative test is required. Tafenoquine should be administered only in patients with an enzymatic activity greater than 70%. Patient counseling ought to also address hemolysis symptoms like fatigue, dyspnea jaundice, and dark-colored urine emission [18].

## 2. Case Report

### 2.1. Patient Background

A 41-year-old male patient from Romania, residing in an urban area and employed as an air traffic controller, presented with no personal or family history of chronic diseases. His only notable medical history was a unilateral orchiectomy. He reported no use of illicit drugs, tobacco, or alcohol, and had no known drug allergies.

### 2.2. Travel History

Prior to admission, the patient had spent 25 days traveling through Southeast Asia without taking malaria chemoprophylaxis. He visited several countries, with four main destinations: Waisai (Indonesia) for 7 days, Dempasar (Bali) for 4 days, and El Nido and Cebu (Philippines) for a total of 9 days.

### 2.3. First Admission

Symptoms began approximately 5 days before hospital admission. The patient initially experienced fever, chills, myalgias, arthralgias, and headache with retro-orbital pain. Within the next 11 h, he developed a dry, irritating cough, followed by vomiting, abdominal pain, and anorexia. At home, he self-administered acetaminophen and amoxicillin–clavulanic acid (a total of four doses), but his symptoms continued to worsen. After 3 days, he sought medical attention at the emergency department and was subsequently referred to the Infectious Diseases Clinic for further evaluation and admitted for investigation.

Upon admission, the patient was in fair general condition and afebrile, with a weight of 76 kg, height of 183 cm, and BMI of 22.69 kg/m^2^. The initial physical examination revealed mild signs of dehydration, a saburral tongue, an erythematous pharynx, hypertrophic tonsils, and jaundice. Cardiovascular and respiratory examinations were unremarkable: the patient had a regular heart rate of 84 bpm, no pathologic murmurs, and clear lung fields without abnormal breath sounds. Oxygen saturation was 98% on room air. No lymphadenopathy was noted. The abdomen was soft and non-tender, with no palpable hepatosplenomegaly. The renal areas were non-tender, and Giordano’s sign was negative bilaterally. Neurological examination revealed slight dysmetria and a mildly positive Romberg test, but no focal neurological deficits or signs of meningeal irritation.

Initial laboratory investigations revealed leukopenia with lymphopenia and severe thrombocytopenia, and a mild inflammatory response with elevated fibrinogen and erythrocyte sedimentation rate (ESR), and a positive latex C-reactive protein (CRP). Liver function tests were within normal limits, but total bilirubin was elevated (Table 1). Rapid diagnostic tests (RDTs) ruled out influenza A/B and SARS-CoV-2 infection. Serology was negative for hepatitis B surface antigen (HBsAg), hepatitis C virus (anti-HCV), and HIV (antigen and antibodies). Blood and urine cultures were also negative.

Given the suggestive clinical presentation and recent travel to Southeast Asia, marked by multiple insect bites and the absence of malaria chemoprophylaxis, a malaria RDT was performed, yielding a positive result. A thick blood smear test confirmed the presence of circulating *Plasmodium* spp. parasites (Table 2).

Initial management included doxycycline, analgesics, and supportive care. The patient was subsequently transferred to the Dr. Victor Babeș Clinical Hospital for Infectious and Tropical Diseases in Bucharest, where an RT-PCR that detected the presence of Plasmodium vivax was performed and targeted antimalarial therapy was initiated: doxycycline 100 mg twice daily for 7 days and atovaquone–proguanil 250/100 mg twice daily for 3 days.

The patient showed a favorable clinical response, with complete resolution of symptoms, and was discharged after 9 days of hospitalization.

### 2.4. Second Admission—First Relapse (25 Days Later)

Approximatively 1 month after the initial treatment, the patient experienced a sudden recurrence of symptoms, including fever, chills, headache, loss of appetite, and asthenia. He was readmitted to the Infectious Diseases Clinic for further evaluation.

On physical examination, the patient was again in fair general condition, though he had lost approximately 3 kg (from 76 kg to 73 kg). He presented with a pale, mildly jaundiced facial appearance. No lymphadenopathy was noted. Cardiovascular and respiratory assessments were within normal limits, with stable vital signs: heart rate of 101 bpm and SpO_2_ of 98% on room air. The abdomen was non-tender, but this time the spleen was palpable. Renal and neurological examinations were unremarkable.

**Figure 1:**
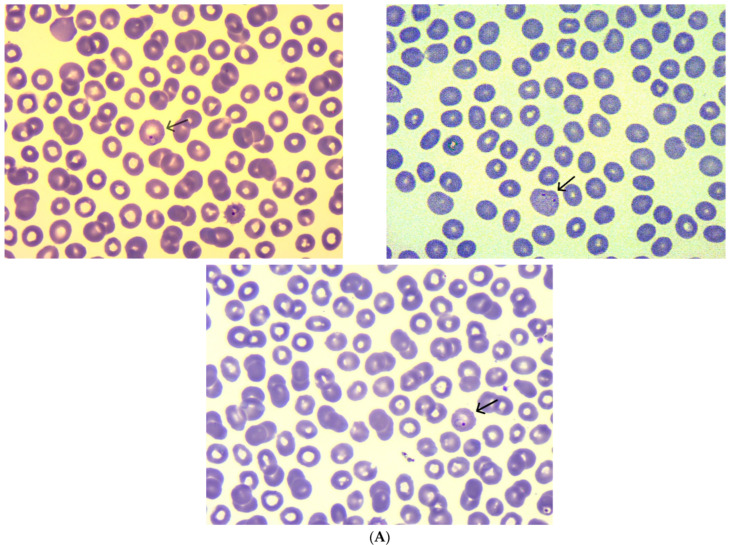

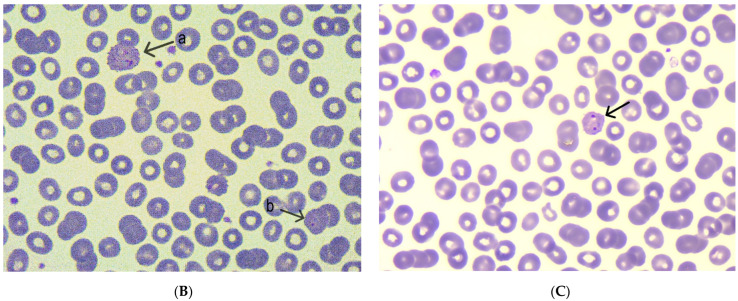
(**A**) Multiple intraerythrocytic forms of *Plasmodium vivax*, including mature ring stages. (**B**,**C**) Representative images showing a *Plasmodium vivax* trophozoite (a) and a mature ring form (b).

Laboratory investigations revealed persistent thrombocytopenia and evidence of systemic inflammation. A cholestatic pattern was observed, along with mild hepatic cytolysis. Electrolyte levels were within normal limits, but creatinine remained constantly near the upper reference limit. RT-PCR tests ruled out COVID-19, influenza, and respiratory syncytial virus. Serologic testing was non-reactive for viral hepatitis markers, including HBsAg, anti-HCV, and anti-HAV (hepatitis A virus) IgM (Table 1). Although the malaria RDT was negative, the patient’s recent history prompted further investigation. A thick blood smear was performed, revealing the presence of *Plasmodium vivax* trophozoites and gametocytes. The diagnosis was confirmed via RT-PCR (Table 2, Figure 1).

The patient was treated with doxycycline 100 mg twice daily for 7 days and atovaquone–proguanil 250/100 mg twice daily for 3 days. Upon discharge, after confirming normal G6PD activity, the patient was prescribed primaquine 15 mg twice daily for 14 days as radical cure therapy.

### 2.5. Third Admission—Second Relapse (60 Days Later)

Two months later, despite completing the full course of primaquine, the patient experienced a second relapse, with mild symptoms, primarily characterized by malaise and asthenia. A peripheral blood smear revealed rare intraerythrocytic forms of *Plasmodium vivax*, and the diagnosis was again confirmed via RT-PCR (Table 2).

The patient was treated with atovaquone–proguanil 250/100 mg (four tablets daily) for 3 consecutive days and doxycycline 100 mg twice daily for 7 days, which led to full clinical recovery.

### 2.6. Follow-Up (After 25 Days)

At the scheduled follow-up, 3 weeks after finishing the antimalarial therapy, the patient reported no fever, headache, or other general or gastrointestinal symptoms.

On clinical examination, he was in good general condition, with no signs of jaundice or pallor. A full laboratory panel was conducted, including complete blood count, inflammatory markers, and renal and liver function tests. All results were within normal reference ranges (Table 1).

The patient made a complete clinical and biological recovery from multiple *Plasmodium vivax* malaria relapses. He was informed about the low risk of further recurrence but advised to seek medical attention should any symptoms reappear. No additional treatment or follow-up was considered necessary.

## 3. Discussion

This case highlights the relapsing nature characteristic of *Plasmodium vivax* malaria and illustrates several key aspects of its epidemiology, its pathophysiology, and the challenges associated with its management.

The patient acquired *Plasmodium vivax* malaria after traveling to Southeast Asia, specifically Indonesia and the Philippines, without taking chemoprophylaxis. This aligns with the known epidemiological patterns of the parasite [14,16]. *Plasmodium vivax* is the most prevalent malaria species outside Africa, accounting for up to 70–90% of malaria cases in Southeast Asia [14,16]. The absence of prophylaxis in this case reflects a common trend observed in imported malaria: most cases in non-endemic regions occur in travelers who either did not take chemoprophylaxis or failed to adhere to the recommended regimen [10,15].

The initial presentation was typical of acute *Plasmodium vivax* malaria, with fever, myalgia, jaundice, and severe thrombocytopenia. Although *Plasmodium vivax* is considered less severe than *Plasmodium falciparum*, it remains a significant cause of morbidity and mortality due to complications such as severe anemia, thrombocytopenia, and splenomegaly [6,14]. According to current guidelines, the diagnosis was appropriately confirmed through a thick blood smear test and a malaria RDT. The use of RT-PCR provided additional sensitivity and specificity, particularly important for detecting low-level parasitemia during relapses [14].

The patient had two distinct relapses, occurring 1 and 2 months after the initial episode, both confirmed by microscopy. A key feature distinguishing *Plasmodium vivax* from other human malaria species is its ability to form dormant liver-stage parasites known as hypnozoites [6,12]. These can reactivate weeks or months after the initial infection, causing relapses in the absence of new exposure. A meta-analysis has estimated that up to 85% of *Plasmodium vivax* reoccurrence cases are due to relapses, rather than reinfection or treatment failure [6,12].

The treatment regimen used, atovaquone–proguanil and doxycycline, is a standard approach for *Plasmodium vivax* malaria, effectively resolving acute symptoms and clearing parasitemia. However, as observed in this case, it did not prevent relapses. In *Plasmodium vivax* malaria, the key principle is that blood-stage antimalarials alone cannot achieve radical cure, as they are not effective against liver-stage hypnozoites [8,14,16]. This explains the occurrence of the first relapse following initial therapy. The administration of primaquine, an antimalarial agent with activity against hypnozoites, typically leads to radical cure when administered correctly to individuals with normal G6PD activity [2,14]. However, the occurrence of the second relapse after primaquine therapy raises important clinical and therapeutic considerations.

When properly administered, primaquine prevents relapses in approximately 80–95% of cases [6,14]. Treatment failures, while uncommon, can occur and may be influenced by several factors. These include suboptimal dosing, particularly in individuals weighing over 60 kg, as the standard 15 mg/day regimen may be insufficient. Some *Plasmodium vivax* strains from Southeast Asia may require higher doses for effective radical cure, possibly due to larger hypnozoite burdens [12,14,17]. Additionally, individual metabolic differences, such as reduced CYP2D6 activity, may lead to treatment failure due to inadequate conversion of primaquine into active metabolites. Poor adherence to therapy is another potential contributor that must be considered [6,12,14], but this topic has a greater impact on endemic countries, where poor adherence is undermining the malaria elimination process. Various mechanisms are involved; for example, incomplete treatment can leave human parasite reservoirs behind, including *Plasmodium vivax* hypnozoites, that can sustain transmission despite having proper vector control. Additionally migrant workers both impair local effectiveness and accelerate the geographic spread of malaria. Also, incomplete or irregular treatment can increase the selection of drug-resistant phenotypes. The importance of proper therapy was highlighted in Costa Rica, where switching to a supervised 7-day treatment scheme with chloroquine and primaquine led to a 98% transmission decline [19].

It is worth mentioning that differentiating between the main causes of malaria reappearing in the same patient is crucial. Firstly, reinfection can be ruled out based on the epidemiology of the disease, because the patient remained in a non-transmission setting without further travel after the treatment, with there being no opportunity for re-exposure to malaria vectors. Studies also observed that treated patients relocated to non-endemic areas still developed recurrent infections that arose from hypnozoites rather than new bites [10,11,14]. Secondly, drug resistance cannot be fully excluded due to the fact that no drug resistance markers were determined, because the stain’s genotyping was not performed. But the fact that the recurrences happened after a symptom-free interval and a longer time-frame than usual for treatment failure, commonly less than 14 days after the initial therapy, argues against recrudescence [11,14,17]. The last option to discuss is relapsing, which is supported by the literature—developing an acute episode more than 21 days after the first episode when the blood-stage drugs were given—aligning with the most common relapse pattern for *Plasmodium vivax* with liver-stage hypnozoites activated between 3 weeks and 3 months post-therapy [11,12,17].

The successful resolution of each acute episode following multiple courses of blood-stage therapy suggests that the patient’s immune system had an effective response, likely developing strain-specific immunity that contributed to milder clinical manifestations during subsequent episodes. The literature supports this observation, indicating that repeated relapses or reinfections with *Plasmodium vivax* can lead to the development of partial clinical immunity, which reduces the frequency and severity of future episodes [1,13,17]. Although the patient made a complete recovery after the final antimalarial course, he should be counseled about the possibility of relapses and instructed to seek prompt medical attention in the event of any febrile illness over the subsequent months.

## 4. Conclusions

This case underscores the complex nature of managing *Plasmodium vivax* malaria, particularly due to its strong tendency to relapse, which can result in multiple episodes of febrile illness, even in non-endemic regions and despite appropriate therapy. The patient’s clinical course, from acquiring the infection in the absence of prophylaxis, through multiple relapses despite radical cure, to eventual resolution, reflects a well-documented pattern associated with *Plasmodium vivax*. This case highlights the importance of accurate etiological diagnosis and the need for prolonged follow-up in patients treated for *Plasmodium vivax* malaria, as relapses can occur weeks or months after the initial episode. It also highlights the importance of pre-travel counseling and the use of chemoprophylaxis when visiting endemic regions.

## Figures and Tables

**Table 1:** Laboratory findings.

Laboratory Test	Normal Range	First Admission	Second Admission	Follow-Up
RBC (10^6^/µL)	4.0–5.5	4.48	4.33	6.64
Hematocrit (%)	40–54	40.2	39.7	42
Hemoglobin (g/dL)	12–16	14.3	13.7	14.4
Lymphocytes # (10^3^/µL)	0.8–4	0.79	0.95	1.82
Lymphocytes %	20–40	20.1	18.2	38.8
Monocytes # (10^3^/µL)	0.12–1.2	0.51	0.4	0.33
Monocytes %	1–10	13	7.7	7
Neutrophils # (10^3^/µL)	1.5–7	2.59	0.95	2.45
Neutrophils %	50–70	65.4	70.7	52.2
Leucocytes (10^3^/µL)	4.0–10	3.95	5.23	4.69
Platelets (10^3^/µL)	150–400	47	100	180
BUN (mg/dL)	19–44	33	31	36
Creatinine (mg/dL)	0.6–1.2	1.34	1.2	1.17
eGFR (mL/min/1.73 m^2^)	>90	65.18	74	–
ALT (U/L)	0–45	26	121	33
AST (U/L)	11–34	30	55	25
Total bilirubin (mg/dL)	0.2–1.2	2.45	3.28	0.65
Direct bilirubin (mg/dL)	0–0.5	0.64	0.88	–
GGT (U/L)	0–55	22	26	–
Serum glucose (mg/dL)	70–105	–	110	86
Amylase (U/L)	28–100	–	45	–
Total proteins (g/L)	60–80	–	75.3	–
Albumin (g/L)	35–52	–	47.7	–
Triglyceride (mg/dL)	0–150	–	193	–
K (mmol/L)	3.5–5.1	3.71	4.34	–
Na (mmol/L)	136–145	132	138	–
Mg (mg/dL)	1.6–2.6	–	1.97	–
Fe (µg/dL)	65–175	–	63	–
CK (U/L)	30–200	82	32	70
Uric acid (mg/dL)	3.7–7.7	–	5.6	–
Alkaline phosphatase (U/L)	50–118	–	124	–
LDH (U/L)	125–220	–	341	146
CRP (mg/dL)	Negative	Positive (>6 mg/dL)	Positive (>6 mg/dL)	–
Fibrinogen (mg/dL)	170–420	374.3	456.7	–
ESR (mm/1 h)	3–10	16	46	6
INR	0.8–1.2	0.94	1.01	–
Procalcitonin	<0.5	<0.5	–	–
Blood culture	Negative	Negative	–	–
Urine culture	<1000 CFU	<1000 CFU	–	–

#–absolute value, RBC–Red blood cell, ALT–Alanine transaminase, AST–Aspartate transaminase, BUN–Blood urea nitrogen, CK–Creatine kinase, CRP–C-reactive protein, EGFR–Estimated glomerular filtration rate, ESR–Erythrocyte sedimentation rate, GGT–Gamma glutamyl transpherase, LDH—Lactate dehydrogenase, INR–International normalized ratio, CFU–Colony-forming units.

**Table 2:** Malaria diagnostic tests.

Test	First Admission	Second Admission	Third Admission
Blood smear microscopy	Normochromic normocytic erythrocytes, discrete poikilocytosis	Normochromic normocytic erythrocytes	Normochromic normocytic erythrocytes
	Presence of *Plasmodium* spp.	Presence of *Plasmodium* spp. with trophozoites and gametocytes	Presence of *Plasmodium* spp.
	Severe thrombocytopenia	Mild thrombocytopenia	Normal thrombocyte count
Parasitemia (%)	–	0.75% parasitemia	0.3% parasitemia
RT-PCR	*Plasmodium vivax* detected	*Plasmodium vivax* detected	–
Malaria RDT	Positive	Negative	Negative

RT-PCR–Reverse-transcription polymerase chain reaction, RDT–Rapid diagnostic tests.

## Data Availability

The data presented in this study are available on request from the corresponding author.

## Abbreviations

RT-PCR	Reverse-transcription polymerase chain reaction
CYP2D6	Cytochrome P450 2D6
G6PD	Glucose-6-phosphate dehydrogenase
BMI	Body-mass index
RDTs	Rapid diagnostic tests
HBsAg	Hepatitis B surface antigen
HCV	Hepatitis C virus
HAV	Hepatitis A virus
RBC	Red blood cell
ALT	Alanine transaminase
AST	Aspartate transaminase
BUN	Blood urea nitrogen
CK	Creatine kinase
CRP	C-reactive protein
EGFR	Estimated glomerular filtration rate
ESR	Erythrocyte sedimentation rate
GGT	Gamma glutamyl transpherase
LDH	Lactate dehydrogenase
INR	International normalized ratio
CFU	Colony-forming units
